# Risk of cancer in patients with glaucoma: A nationwide population-based cohort study

**DOI:** 10.1038/s41598-020-65116-8

**Published:** 2020-05-18

**Authors:** Younhea Jung, Kyungdo Han, Kyung-sun Na, Gee-hyun Kim, Minji Ha, Ji-Sun Paik, Jung Il Moon

**Affiliations:** 1 Department of Ophthalmology, College of Medicine, Yeouido St. Mary’s Hospital, The Catholic University of Korea, Seoul, Republic of Korea; 2 Department of Statistics and Actuarial Science, Soongsil University, Seoul, Republic of Korea

**Keywords:** Optic nerve diseases, Epidemiology

## Abstract

To compare the risk of cancer development between patients with glaucoma and those without, we conducted a nationwide population-based cohort study using the Korean National Health Insurance Database. Individuals with diagnosis of glaucoma between 2007 and 2016 were identified, and controls were 1:1 matched based on age and sex. We calculated the incidence rates(IR) and hazard ratios(HR) before and after adjusting for age, gender, diabetes, smoking history, and body mass index. A total of 107,536 individuals with glaucoma and the same number of individuals without glaucoma were included. The IR of overall cancer were 12.23 and 11.62 per 1,000 individuals in the glaucoma and control groups, respectively. The HR of overall cancer was significantly higher in the glaucoma group before(HR: 1.053) and after adjusting for confounding factors(adjusted HR: 1.049) compared to that in the control group. The risk of overall cancer and specific cancers varied depending on gender and age groups, and the association was stronger in women and those under 65 years of age. Our study revealed that individuals with glaucoma showed higher risk of overall cancer and higher risk of specific cancers than those without glaucoma.

## Introduction

Glaucoma is the leading cause of irreversible blindness worldwide, making it a major public health challenge^1–3^. It is a neurodegenerative disease characterized by progressive retinal ganglion cell death caused by multifactorial etiology including high intraocular pressure (IOP), neurotrophin insufficiency, local ischemia-hypoxia, inflammation, glutamate excitotoxicity, oxidative stress, and aberrant immunity^4–8^.

Among these risk factors, elevated IOP is the major risk factor^1,4^, however, patients with normal-tension glaucoma, the most predominant form of glaucoma in Korea^9^, have normal range IOP. This points to the importance of other mechanisms beyond IOP, such as inflammation in the pathogenesis of glaucoma^7,8,10,11^. Previous studies have shown that inflammatory mediators such as tumor necrosis factor alpha, interleukins, endothelin-1, and C-reactive protein are elevated in glaucoma^12–15^.

Inflammation plays decisive roles at all stages of cancer development^16–18^. Chronic inflammation increases the risk of carcinogenesis, from initiation, promotion, malignant conversion, to metastasis, and even subclinical, low grade inflammation may be as important in promoting cancer. Previous studies have shown strong and recurrent associations between inflammatory bowel diseases and cancer, not just limited to gut malignancies but also other sites including lung, kidney, endocrine, cervix, and prostate^19,20^. Increased risk of overall cancer and various focal malignancies has been reported in patients with rheumatoid arthritis and systemic lupus erythematosus (SLE)^21,22^.

In light with this, it is possible that glaucoma may contribute to increased risk of cancer, however, the association of the two disease entities has not yet been investigated. Identifying the risk of overall cancer and specific types of cancer in glaucoma patients may improve our understanding of both diseases and provide guidelines for appropriate diagnostic work-up for cancer screening in glaucoma patients.

Therefore, in this nationwide, population-based, age- and sex-matched cohort study, we aimed to investigate the risk of cancer development in patients with glaucoma using the National Health Insurance Database (NHID) provided by the Korean National Health Insurance Service (KNHIS), which covers almost the entire 50 million Korean population.

## Results

In this study, 107,536 individuals with glaucoma and the same number of individuals without glaucoma matched for age and sex were included (Supplementary Fig. 1). Table 1 describes the baseline characteristics of the study population. The mean age of the study population was 62.33 ± 10.89 years, and 54,621 (50.79%) were men. The glaucoma group tended to live in the rural area (*P* < 0.001) and have more frequent diabetes (*P* < 0.001), hypertension (*P* < 0.001), and dyslipidemia (*P* < 0.001) than the control group. They also showed significantly lower cholesterol level (*P* < 0.001), higher glucose (*P* < 0.001), and lower systolic and diastolic blood pressure (*P* < 0.001, both) compared to controls. The mean follow-up time was 4.63 ± 2.50 years and 4.64 ± 2.51 years for individuals with and without glaucoma, respectively (*P* = 0.152).

**Table 1:** Baseline characteristics of the study population.

Parameters	Control	Glaucoma	*P* value
	*N* = 107536	*N* = 107536	
Age, years	62.33 ± 10.89	62.33 ± 10.89	1
Age groups			1
40–49	15533(14.44)	15533(14.44)	
50–59	25944(24.13)	25944(24.13)	
60–69	33214(30.89)	33214(30.89)	
70–79	27870(25.92)	27870(25.92)	
80-	4975(4.63)	4975(4.63)	
Gender			1
Male	54621(50.79)	54621(50.79)	
Female	52915(49.21)	52915(49.21)	
Income			0.2697
Lowest quartile	23140(21.52)	22930(21.32)	
Remaining quartiles	84396(78.48)	84606(78.68)	
Residential area			<0.0001
Urban	46818(43.54)	50478(46.94)	
Rural	60718(56.46)	57058(53.06)	
Diabetes mellitus			<0.0001
No	89959(83.65)	82029(76.28)	
Yes	17577(16.35)	25507(23.72)	
Hypertension			<0.0001
No	58468(54.37)	53146(49.42)	
Yes	49068(45.63)	54390(50.58)	
Dyslipidemia			<0.0001
No	77493(72.06)	70228(65.31)	
Yes	30043(27.94)	37308(34.69)	
Smoking history			<0.0001
No	71102(66.12)	72340(67.27)	
Ex-smoker	17988(16.73)	20330(18.91)	
Current smoker	18446(17.15)	14866(13.82)	
Body mass index	24.07 ± 3.09	24.05 ± 3.07	0.0659
Total cholesterol	197.01 ± 38.18	194.97 ± 39.13	<0.0001
Systolic blood pressure	126.62 ± 15.56	126.33 ± 15.63	<0.0001
Diastolic blood pressure	77.47 ± 9.96	77.16 ± 9.96	<0.0001
Fasting glucose	102.43 ± 25.82	106 ± 30.41	<0.0001

### Risk of cancer in glaucoma

**Figure 1:**
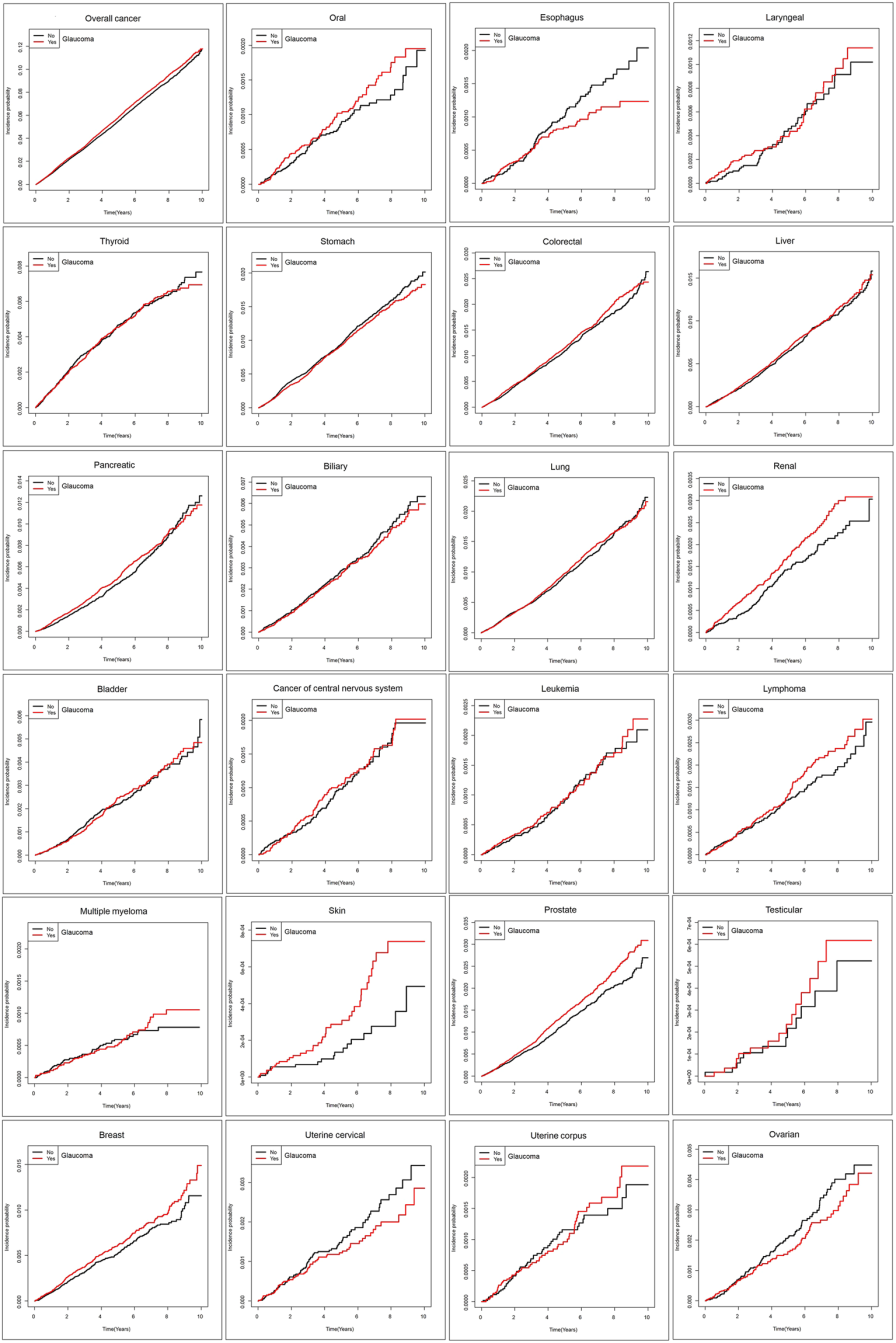
Cumulative incidence probability of overall and specific cancers in control and glaucoma groups.

The number of newly diagnosed cancer and incidence rates are presented in Table 2. The cumulative incidence probability over time is presented in Fig. 1. The total cancer incidence rate was 12.23 per 1,000 persons in the glaucoma group, which was higher than 11.62 per 1,000 persons in the matched control group. The HR for overall cancer was significantly higher in the glaucoma group before (HR: 1.053, 95% CI: 1.015–1.091) and after adjusting for age, gender, diabetes, smoking history, and body mass index (adjusted HR: 1.049, 95% CI: 1.012–1.088) compared to that in the control group. Individuals with glaucoma exhibited increased risk of skin cancer before (HR: 2.003, 95% CI: 1.138–3.527) and after adjusting for confounding factors (adjusted HR: 1.944, 95% CI: 1.102–3.430); however, the confidence interval was large due to limited number of events. Risk of prostate cancer was also increased in the glaucoma group prior to (HR: 1.174, 95% CI: 1.055–1.307) and after adjustment (adjusted HR: 1.168, 95% CI: 1049–1.300).

**Table 2:** Comparison of incidence rates and hazard ratios for overall and specific cancers between control and glaucoma groups.

	Control (*N* = 107536)		Glaucoma (*N* = 107536)		Hazard ratio (95% confidence interval)		
	N. of events	Incidence rate (per 1000)	N. of events	Incidence rate (per 1000)	Model 1	Model 2	Model 3
Overall cancer	5802	11.617	6086	12.227	**1.053** (1.015, 1.091)*	**1.051** (1.014, 1.09)*	**1.049** (1.012, 1.088)*
Oral	89	0.174	106	0.207	1.192 (0.899, 1.58)	1.191 (0.898, 1.578)	1.217 (0.917, 1.616)
Esophagus	103	0.201	81	0.158	0.787 (0.588, 1.053)	0.784 (0.586, 1.049)	0.805 (0.6, 1.079)
Laryngeal	48	0.094	53	0.104	1.105 (0.748, 1.634)	1.102 (0.746, 1.629)	1.147 (0.773, 1.7)
Thyroid	456	0.892	447	0.876	0.981 (0.861, 1.118)	0.982 (0.862, 1.118)	0.997 (0.874, 1.137)
Stomach	1015	1.990	954	1.872	0.941 (0.861, 1.028)	0.938 (0.859, 1.025)	0.935 (0.856, 1.022)
Colorectal	1162	2.279	1227	2.410	1.058 (0.976, 1.146)	1.056 (0.974, 1.144)	1.04 (0.959, 1.127)
Liver	679	1.327	707	1.383	1.043 (0.938, 1.158)	1.04 (0.936, 1.156)	1.016 (0.914, 1.13)
Pancreatic	505	0.986	545	1.066	1.081 (0.958, 1.22)	1.078 (0.955, 1.217)	1.055 (0.934, 1.191)
Biliary	300	0.586	285	0.557	0.951 (0.809, 1.118)	0.947 (0.806, 1.114)	0.935 (0.795, 1.101)
Lung	982	1.921	1007	1.972	1.027 (0.94, 1.121)	1.023 (0.937, 1.117)	1.059 (0.97, 1.157)
Renal	139	0.271	178	0.348	1.282 (1.027, 1.601)	1.281 (1.026, 1.599)	1.239 (0.991, 1.549)
Bladder	233	0.455	234	0.457	1.005 (0.839, 1.205)	1 (0.834, 1.199)	0.998 (0.831, 1.198)
Cancer of central nervous system	102	0.199	108	0.211	1.06 (0.809, 1.39)	1.059 (0.808, 1.388)	1.074 (0.818, 1.409)
Leukemia	98	0.191	102	0.199	1.042 (0.79, 1.375)	1.04 (0.788, 1.373)	1.03 (0.779, 1.361)
Lymphoma	126	0.246	146	0.285	1.16 (0.914, 1.472)	1.158 (0.912, 1.469)	1.19 (0.937, 1.513)
Multiple myeloma	57	0.111	60	0.117	1.054 (0.733, 1.514)	1.051 (0.731, 1.51)	1.033 (0.718, 1.488)
Skin	18	0.035	36	0.070	**2.003** (**1.138**, 3.527)*	**2** (**1.136**, 3.521)*	**1.944** (**1.102**, 3.43)*
Prostate	625	2.427	732	2.849	**1.174** (**1.055**, 1.307)*	**1.17** (**1.052**, 1.302)*	**1.168** (**1.049**, 1.3)*
Testicular	12	0.046	15	0.058	1.252 (0.586, 2.676)	1.25 (0.585, 2.67)	1.289 (0.6, 2.768)
Breast	278	1.099	324	1.284	1.167 (0.995, 1.37)	1.169 (0.996, 1.372)	1.168 (0.994, 1.372)
Uterine cervical	82	0.324	67	0.265	0.818 (0.592, 1.13)	0.818 (0.592, 1.129)	0.814 (0.588, 1.126)
Uterine corpus	53	0.209	56	0.221	1.058 (0.726, 1.54)	1.058 (0.727, 1.54)	1.051 (0.72, 1.535)
Ovarian	111	0.438	93	0.367	0.839 (0.637, 1.105)	0.839 (0.637, 1.105)	0.845 (0.64, 1.115)

Model 1 Unadjusted.

Model 2 Adjusted for age and gender.

Model 3 Adjusted for age, gender, diabetes, smoking history, and body mass index.

Female malignancies are analyzed in females(n = 52915).

Male malignancies are analyzed in males (n = 54621).

^*^P value < 0.05.

### Risk of cancer according to gender

The number of cancer events and incidence rates in males and females are shown in Tables 3 and 4, respectively. In men, those with glaucoma were more likely to develop oral (adjusted HR: 1.491, 95% CI: 1.052–2.114), skin (adjusted HR: 4.42, 95% CI: 1.493–13.086), and prostate (adjusted HR: 1.168, 95% CI: 1.049–1.300) cancers, but less likely to develop biliary cancer (adjusted HR: 0.796, 95% CI: 0.639–0.993) than those without glaucoma.

**Table 3:** Comparison of incidence rates and hazard ratios for overall and specific cancers between control and glaucoma groups in men.

	Control (*N* = 54621)		Glaucoma (*N* = 54621)		Hazard ratio (95% confidence interval)		
	N. of events	Incidence rate (per 1000)	N. of events	Incidence rate (per 1000)	Model 1	Model 2	Model 3
Overall cancer	3582	14.256	3713	14.832	1.041(0.994, 1.09)	1.038 (0.992, 1.087)	1.039 (0.992, 1.088)
Oral	54	0.209	79	0.305	1.465 (1.036, 2.07)*	1.463 (1.035, 2.067)*	1.491 (1.052, 2.114)*
Esophagus	96	0.371	76	0.294	0.792 (0.586, 1.071)	0.789 (0.584, 1.066)	0.811 (0.599, 1.098)
Laryngeal	44	0.170	50	0.193	1.138 (0.759, 1.706)	1.135 (0.757, 1.701)	1.178 (0.783, 1.773)
Thyroid	104	0.402	115	0.445	1.107 (0.849, 1.443)	1.108 (0.85, 1.444)	1.091 (0.835, 1.425)
Stomach	729	2.834	681	2.651	0.935(0.843, 1.038)	0.933(0.84, 1.035)	0.934(0.841, 1.038)
Colorectal	721	2.802	710	2.762	0.986(0.889, 1.093)	0.983(0.886, 1.09)	0.967(0.871, 1.073)
Liver	487	1.885	498	1.930	1.024(0.904, 1.16)	1.022 (0.902, 1.158)	0.993 (0.876, 1.126)
Pancreatic	300	1.160	316	1.223	1.055 (0.901, 1.235)	1.051 (0.898, 1.231)	1.026 (0.875, 1.203)
Biliary	180	0.695	144	0.557	0.801 (0.643, 0.997)*	0.797 (0.64, 0.992)*	0.796 (0.639, 0.993)*
Lung	746	2.890	713	2.765	0.957 (0.864, 1.06)	0.952 (0.859, 1.055)	0.995 (0.898, 1.104)
Renal	100	0.386	120	0.464	1.201 (0.921, 1.567)	1.2 (0.921, 1.565)	1.132 (0.866, 1.479)
Bladder	191	0.739	198	0.766	1.038 (0.851, 1.266)	1.032 (0.846, 1.259)	1.029 (0.842, 1.257)
Cancer of central nervous system	55	0.212	56	0.216	1.02 (0.703, 1.479)	1.016 (0.701, 1.474)	1.054 (0.725, 1.533)
Leukemia	55	0.212	61	0.236	1.111 (0.771, 1.599)	1.108 (0.77, 1.595)	1.095 (0.759, 1.58)
Lymphoma	87	0.336	85	0.329	0.978 (0.725, 1.318)	0.975 (0.723, 1.314)	1.013 (0.75, 1.369)
Multiple myeloma	30	0.116	37	0.143	1.235 (0.763, 1.999)	1.229 (0.76, 1.99)	1.189 (0.733, 1.93)
Skin	4	0.015	18	0.070	4.511 (1.527, 13.328)*	4.5 (1.523, 13.296)*	4.42 (1.493, 13.086)*
Prostate	625	2.427	732	2.849	1.174 (1.055, 1.307)*	1.17 (1.052, 1.302)*	1.168 (1.049, 1.3)*
Testicular	12	0.046	15	0.058	1.252 (0.586, 2.676)	1.25 (0.585, 2.67)	1.289 (0.6, 2.768)

Model 1 Unadjusted.

Model 2 Adjusted for age.

Model 3 Adjusted for age, diabetes, smoking history, and body mass index.

*P value < 0.05.

**Table 4:** Comparison of incidence rates and hazard ratios for overall and specific cancers between control and glaucoma groups in women.

	Control (*N* = 52915)		Glaucoma (*N* = 52915)		Hazard ratio (95% confidence interval)		
	N. of events	Incidence rate (per 1000)	N. of events	Incidence rate (per 1000)	Model 1	Model 2	Model 3
Overall cancer	2220	8.946	2373	9.591	1.072 (1.012, 1.136)*	1.072 (1.011, 1.135)*	1.07 (1.009, 1.134)*
Oral	35	0.138	27	0.107	0.772(0.467, 1.275)	0.771(0.466, 1.273)	0.791(0.478, 1.308)
Esophagus	7	0.028	5	0.020	0.715(0.227, 2.253)	0.711(0.226, 2.241)	0.702(0.222, 2.217)
Laryngeal	4	0.016	3	0.012	0.75 (0.168, 3.353)	0.748 (0.167, 3.342)	0.795 (0.177, 3.571)
Thyroid	352	1.395	332	1.317	0.944 (0.812, 1.096)	0.944 (0.813, 1.097)	0.969 (0.833, 1.127)
Stomach	286	1.131	273	1.080	0.955 (0.809, 1.127)	0.953 (0.808, 1.125)	0.94 (0.796, 1.111)
Colorectal	441	1.746	517	2.051	1.175 (1.034, 1.334)*	1.174 (1.034, 1.333)*	1.162 (1.023, 1.32)*
Liver	192	0.758	209	0.826	1.09 (0.896, 1.326)	1.088 (0.894, 1.323)	1.079 (0.886, 1.313)
Pancreatic	205	0.809	229	0.905	1.119 (0.926, 1.351)	1.117 (0.925, 1.349)	1.099 (0.909, 1.328)
Biliary	120	0.474	141	0.557	1.176 (0.922, 1.5)	1.173 (0.92, 1.497)	1.142 (0.894, 1.458)
Lung	236	0.932	294	1.163	1.248 (1.051, 1.481)*	1.246 (1.05, 1.479)*	1.264 (1.064, 1.501)*
Renal	39	0.154	58	0.229	1.489 (0.992, 2.235)*	1.488 (0.992, 2.233)*	1.519 (1.01, 2.283)*
Bladder	42	0.166	36	0.142	0.858 (0.55, 1.34)	0.856 (0.548, 1.335)	0.856 (0.548, 1.339)
Cancer of central nervous system	47	0.185	52	0.205	1.107 (0.746, 1.643)	1.107 (0.746, 1.642)	1.105 (0.743, 1.643)
Leukemia	43	0.170	41	0.162	0.954 (0.622, 1.464)	0.953 (0.621, 1.462)	0.952 (0.619, 1.463)
Lymphoma	39	0.154	61	0.241	1.566 (1.048, 2.341)*	1.565 (1.047, 2.339)*	1.582 (1.057, 2.368)*
Multiple myeloma	27	0.107	23	0.091	0.853 (0.489, 1.488)	0.852 (0.489, 1.486)	0.863 (0.494, 1.51)
Skin	14	0.055	18	0.071	1.287 (0.64, 2.588)	1.285 (0.639, 2.583)	1.231 (0.611, 2.483)
Breast	278	1.099	324	1.284	1.167 (0.995,1.37)	1.169 (0.996,1.372)	1.168 (0.994,1.372)
Uterine cervical	82	0.324	67	0.265	0.818 (0.592, 1.13)	0.818 (0.592, 1.129)	0.814 (0.588, 1.126)
Uterine corpus	53	0.209	56	0.221	1.058 (0.726, 1.54)	1.058 (0.727, 1.54)	1.051 (0.72, 1.535)
Ovarian	111	0.438	93	0.367	0.839 (0.637, 1.105)	0.839 (0.637, 1.105)	0.845 (0.64, 1.115)

Model 1 Unadjusted.

Model 2 Adjusted for age.

Model 3 Adjusted for age, diabetes, smoking history, and body mass index.

*P value < 0.05.

Meanwhile, women with glaucoma had a greater risk for overall cancer (adjusted HR: 1.070, 95% CI: 1.009–1.134), colorectal (adjusted HR: 1.162, 95% CI:1.023–1.320), lung (adjusted HR: 1.264, 95% CI: 1.064–1.501), and renal (adjusted HR: 1.519, 95% CI: 1.010–2.283) cancers and lymphoma (adjusted HR: 1.582, 95% CI: 1.057–2.368) compared to those without glaucoma.

### Risk of cancer according to age group

Younger patients (age <65 years) with glaucoma had a greater risk of overall cancer (adjusted HR: 1.086, 95% CI: 1.023–1.152) compared with those without glaucoma (Table 5). They showed increased risk of renal (adjusted HR: 1.462, 95% CI: 1.036–2.063), skin (adjusted HR: 3.437, 95% CI:1.125–10.498) and prostate (adjusted HR: 1.259, 95% CI: 1.022–1.552) cancers, but decreased risk of esophageal (adjusted HR: 0.564, 95% CI: 0.320–0.994) cancer.

**Table 5:** Comparison of incidence rates and hazard ratios for overall and specific cancers between control and glaucoma groups in those <65 years.

	Control (*N* = 59567)		Glaucoma (*N* = 59567)		Hazard ratio (95% confidence interval)		
	N. of events	Incidence rate (per 1000)	N. of events	Incidence rate (per 1000)	Model 1	Model 2	Model 3
Overall cancer	2140	7.496	2321	8.166	1.09 (1.027, 1.156)*	1.09 (1.027, 1.156)*	1.086 (1.023, 1.152)*
Oral	36	0.124	39	0.134	1.086 (0.69, 1.708)	1.086 (0.69, 1.708)	1.089 (0.689, 1.723)
Esophagus	35	0.120	19	0.065	0.544 (0.311, 0.951)*	0.544 (0.311, 0.951)*	0.564 (0.32, 0.994)*
Laryngeal	15	0.052	24	0.083	1.605 (0.842, 3.059)	1.604 (0.842, 3.058)	1.705 (0.885, 3.285)
Thyroid	326	1.124	334	1.155	1.027 (0.881, 1.196)	1.027 (0.882, 1.196)	1.05 (0.901, 1.225)
Stomach	350	1.206	363	1.254	1.04 (0.898, 1.204)	1.04 (0.898, 1.205)	1.021 (0.88, 1.185)
Colorectal	417	1.438	408	1.410	0.981 (0.856, 1.124)	0.981 (0.856, 1.124)	0.964 (0.84, 1.107)
Liver	236	0.812	261	0.900	1.109 (0.93, 1.322)	1.109 (0.93, 1.322)	1.074 (0.898, 1.283)
Pancreatic	163	0.560	176	0.606	1.083 (0.875, 1.34)	1.083 (0.875, 1.341)	1.035 (0.834, 1.284)
Biliary	76	0.261	82	0.282	1.081 (0.792, 1.477)	1.082 (0.792, 1.478)	1.057 (0.771, 1.448)
Lung	262	0.901	290	1.000	1.11 (0.939, 1.312)	1.11 (0.939, 1.312)	1.154 (0.975, 1.366)
Renal	55	0.189	83	0.286	1.513 (1.076, 2.128)*	1.514 (1.077, 2.128)*	1.462 (1.036, 2.063)*
Bladder	53	0.182	59	0.203	1.116 (0.77, 1.617)	1.116 (0.77, 1.617)	1.117 (0.767, 1.625)
Cancer of central nervous system	43	0.148	39	0.134	0.909 (0.59, 1.403)	0.91 (0.59, 1.403)	0.919 (0.594, 1.424)
Leukemia	35	0.120	36	0.124	1.032 (0.648, 1.643)	1.032 (0.648, 1.643)	1.018 (0.636, 1.629)
Lymphoma	52	0.179	51	0.176	0.983 (0.668, 1.447)	0.984 (0.668, 1.447)	1.015 (0.687, 1.498)
Multiple myeloma	16	0.055	24	0.083	1.503 (0.799, 2.83)	1.504 (0.799, 2.831)	1.489 (0.787, 2.818)
Skin	4	0.014	14	0.048	3.509 (1.155, 10.661)*	3.511 (1.156, 10.666)*	3.437 (1.125, 10.498)*
Prostate^†^	162	1.002	201	1.245	1.244 (1.011, 1.53)*	1.245 (1.012, 1.531)*	1.259 (1.022, 1.552)*
Testicular^†^	3	0.018	8	0.049	2.674 (0.709, 10.079)	2.675 (0.71, 10.083)	2.903 (0.761, 11.072)
Breast^‡^	182	1.416	209	1.632	1.153 (0.945, 1.406)	1.153 (0.945, 1.406)	1.156 (0.946, 1.413)
Uterine cervical^‡^	32	0.248	30	0.233	0.94 (0.571, 1.547)	0.94 (0.571, 1.548)	0.936 (0.566, 1.548)
Uterine corpus^‡^	30	0.233	40	0.311	1.338 (0.833, 2.147)	1.337 (0.833, 2.147)	1.36 (0.843, 2.191)
Ovarian^‡^	58	0.450	53	0.412	0.917 (0.632, 1.331)	0.917 (0.632, 1.331)	0.914 (0.628, 1.332)

Model 1 Unadjusted.

Model 2 Adjusted for age and gender.

Model 3 Adjusted for age, gender, diabetes, smoking history, and body mass index.

*P value < 0.05.

^†^Male malignancies are analyzed in males (n = 33247).

^‡^Female malignancies are analyzed in females (n = 26320).

Older patients aged 65 years or older with glaucoma exhibited increased hazard of developing prostate cancer (adjusted HR: 1.150, 95% CI: 1.015–1.304), but decreased hazard of stomach cancer (adjusted HR: 0.892, 95% CI: 0.798–0.997) incidence (Table 6).

**Table 6:** Comparison of incidence rates and hazard ratios for overall and specific cancers between control and glaucoma groups in those ≥65 years.

	Control (*N* = 47969)		Glaucoma (*N* = 47969)		Hazard ratio (95% confidence interval)		
	N. of events	Incidence rate (per 1000)	N. of events	Incidence rate (per 1000)	Model 1	Model 2	Model 3
Overall cancer	3662	17.117	3765	17.632	1.03 (0.984, 1.078)	1.03 (0.984, 1.078)	1.032 (0.986, 1.08)
Oral	53	0.239	67	0.303	1.264 (0.881, 1.812)	1.263 (0.881, 1.811)	1.298 (0.904, 1.865)
Esophagus	68	0.307	62	0.280	0.911 (0.646, 1.285)	0.91 (0.645, 1.284)	0.933 (0.66, 1.319)
Laryngeal	33	0.149	29	0.131	0.878 (0.533, 1.446)	0.879 (0.533, 1.447)	0.909 (0.55, 1.501)
Thyroid	130	0.588	113	0.511	0.869 (0.675, 1.118)	0.869 (0.675, 1.118)	0.877 (0.681, 1.129)
Stomach	665	3.025	591	2.685	**0.888** (**0.794**, 0.992)*	**0.887** (**0.794**, 0.99)*	**0.892** (**0.798**, 0.997)*
Colorectal	745	3.390	819	3.729	1.1 (0.996, 1.215)	1.099 (0.995, 1.214)	1.085 (0.982, 1.199)
Liver	443	2.005	446	2.018	1.006 (0.882, 1.148)	1.005 (0.881, 1.147)	0.989 (0.866, 1.128)
Pancreatic	342	1.547	369	1.669	1.079 (0.931, 1.25)	1.078 (0.93, 1.249)	1.064 (0.918, 1.234)
Biliary	224	1.013	203	0.917	0.905 (0.749, 1.095)	0.904 (0.747, 1.093)	0.898 (0.742, 1.087)
Lung	720	3.264	717	3.249	0.995 (0.898, 1.104)	0.994 (0.896, 1.102)	1.033 (0.931, 1.146)
Renal	84	0.380	95	0.429	1.13 (0.843, 1.516)	1.13 (0.843, 1.516)	1.1 (0.819, 1.477)
Bladder	180	0.814	175	0.791	0.971 (0.789, 1.196)	0.968 (0.786, 1.192)	0.969 (0.786, 1.195)
Cancer of central nervous system	59	0.267	69	0.312	1.169 (0.826, 1.654)	1.168 (0.825, 1.653)	1.19 (0.839, 1.687)
Leukemia	63	0.285	66	0.298	1.047 (0.741, 1.479)	1.047 (0.741, 1.479)	1.042 (0.737, 1.474)
Lymphoma	74	0.334	95	0.429	1.283 (0.947, 1.739)	1.281 (0.945, 1.736)	1.319 (0.972, 1.79)
Multiple myeloma	41	0.185	36	0.162	0.877 (0.561, 1.373)	0.874 (0.559, 1.368)	0.86 (0.549, 1.349)
Skin	14	0.063	22	0.099	1.571 (0.804, 3.071)	1.569 (0.803, 3.066)	1.527 (0.78, 2.99)
Prostate^†^	463	4.833	531	5.560	**1.151** (**1.016**, 1.304)*	**1.149** (**1.014**, 1.301)*	**1.15** (**1.015**, 1.304)*
Testicular^†^	9	0.093	7	0.072	0.778 (0.29, 2.09)	0.779 (0.29, 2.092)	0.788 (0.292, 2.125)
Breast^‡^	96	0.772	115	0.925	1.197 (0.913, 1.569)	1.198 (0.914, 1.571)	1.202 (0.915, 1.577)
Uterine cervical^‡^	50	0.402	37	0.297	0.739 (0.483, 1.131)	0.739 (0.483, 1.131)	0.739 (0.482, 1.132)
Uterine corpus^‡^	23	0.185	16	0.128	0.695 (0.367, 1.315)	0.695 (0.367, 1.315)	0.674 (0.355, 1.28)
Ovarian^‡^	53	0.426	40	0.321	0.754 (0.5, 1.136)	0.754 (0.5, 1.136)	0.762 (0.505, 1.15)

Model 1 Unadjusted.

Model 2 Adjusted for age and gender.

Model 3 Adjusted for age, gender, diabetes, smoking history, and body mass index.

*P value < 0.05.

^†^Male malignancies are analyzed in males(n = 21374).

^‡^Female malignancies are analyzed in females(n = 26595).

## Discussion

In this nationwide population-based study, we compared the incidence of overall cancer and various specific cancers between 107,536 glaucoma subjects and age- and sex- matched controls. We found that individuals with glaucoma showed significantly higher risk of developing cancer overall and specific cancers including prostate and skin than those without glaucoma before and after adjusting for confounding factors. To the best of our knowledge, this is the first study that has assessed the association between glaucoma and cancer incidence.

The risk of cancer in specific organs varied depending on gender and age groups, and the association was stronger in women and those under 65 years of age. In males, the risk of oral, skin, and prostate cancers increased and that of biliary cancer decreased compared to the age-matched control group. However, in females, individuals with glaucoma had higher risk of overall cancer as well as colorectal, lung, and renal cancers and lymphoma compared to the matched control group.

After stratifying by age, in those under the age of 65, individuals with glaucoma showed increased risk of overall, renal, skin, and prostate cancers and decreased risk of esophageal cancer. In individuals aged 65 years or older, those with glaucoma exhibited higher risk of prostate cancer. However, the risk of stomach cancer compared to matched controls was lower, which was unexpected, because significant association between H. Pylori infection, one of the major risk factors for stomach cancer, and glaucoma has been reported in previous studies^23^.

Although the reason for the association between glaucoma and various cancers is not well known, there are several potential mechanisms. First, the most important risk factor for glaucoma is elevated intraocular pressure, but there are other important factors including inflammation, which may link glaucoma with cancer. Inflammation plays a role in the pathogenesis of glaucoma, in which low grade chronic inflammation occurs in response to elevated intraocular pressure and vascular dysfunction^24,25^. In healthy eyes, the blood-ocular barrier prevents many molecular substances from crossing from the blood into the eye and vice versa, but in glaucomatous eyes, this barrier around the optic nerve head has been shown to leak^26^. Furthermore, inflammatory molecules including tumor necrosis factor alpha, interleukins, endothelin-1, C-reactive protein, vascular endothelial growth factor, and cyclooxygenase-2/prostaglandin E2 have been reported to be elevated in glaucoma^12,15,27^.

Inflammation predisposes to cancer development and plays critical roles at all stages of tumorigenesis from tumor initiation, growth, and progression, to metastasis^18,28^. Chronic systemic inflammation in glaucoma may contribute to enhanced carcinogenesis through inflammation-related mechanisms^16,29,30^. Other inflammatory diseases such as inflammatory bowel diseases^19^, rheumatoid arthritis^31^, systemic lupus arthritis^21^, and type 1 diabetes mellitus^32^ have been reported to increase the risk of cancer. In individuals with rheumatoid arthritis, the risk of overall cancer and the risk of specific cancers including lymphoma, larynx, oropharynx, esophagus, lung, kidney, bladder, prostate, liver, skin, and pancreas was increased^33^. In patients with SLE, the risk of overall cancer and also of focal malignancies such as lung, kidney, bladder, esophagus, stomach, colon, liver, and pancreas was shown to be increased. [21 Specific cancers related with these inflammatory diseases are similar to our results.

There are also environmental factors. Smoking may be another link between glaucoma and cancer. There are numerous studies reporting that smoking is associated with higher risk of glaucoma^34,35^. In our study, specific cancers shown to be increased in patients with glaucoma are mostly smoking-related cancers, which include lung, oral, renal, and colorectal^36^. However, the association between glaucoma and cancer was significant even after adjusting for smoking history, and other smoking-related cancers such as esophageal and gastric cancers showed decreased risk in patients with glaucoma, so the results of this study cannot be explained by smoking alone.

Furthermore, our findings could also be partly explained by the hypothesis that patients with glaucoma are expected to visit physicians more often and may show different health screening behavior compared to those without glaucoma. They may be more likely to receive screening for other diseases, leading to more frequent diagnosis of certain types of malignancies. On the other hand, this may also lead to lower incidence of certain tumors, for instance, they may receive endoscopy more often, enabling treatment of precancer lesions which may account for the lower risk of stomach cancer in the older group.

This study has several strengths. First, a cohort study using a large database such as the Korean NHID would be optimal in assessing the association between glaucoma and cancers. Randomized clinical trials would not be feasible, and case-cohort studies would be influenced by survival effects, indicating that if glaucoma-related mortalities occurred before the cancer diagnosis, the risk of cancer would be underestimated. Second, this nationwide population-based longitudinal database enables accurate follow up of medical records of almost the entire 50 million population in Korea, allowing evaluation of temporal association between glaucoma and various cancers. Furthermore, we also included results from the general health screening examinations, which enabled adjustments for various parameters such as body mass index.

There are still limitations of this study. First, glaucoma was defined based on medical records, indicating those who did not seek medical service were not included in the study underestimating the glaucoma population. However, it is likely that this would underestimate the HR. In addition, we could not perform further analysis on the risk of cancers in different types of glaucoma. Further studies are warranted to explore this relationship. Furthermore, although the cancer incidence that we report in this study is based on one of the largest cohorts of glaucoma, this study only included Koreans. As different ethnicity and environmental exposures affect disease pathogenesis, future studies are needed in different ethnic groups.

In conclusion, based on this nationwide longitudinal study investigating the association between glaucoma and cancer development, we found that glaucoma is a risk factor for overall cancer incidence. In addition, the risk of cancer was more prominent in female glaucoma patients and those younger than 65 years. Considering the globally growing number of individuals with glaucoma, healthcare providers should be aware of the increased risk of cancer in individuals with glaucoma.

## Methods

### National Health Insurance Database and national general health screening in Korea

In this nationwide, population-based cohort study, we used the NHID provided by the KNHIS, which mandates all nationals to enroll in the system. The KNHIS covers about 97% of the population and provides universal health coverage. The NHID includes information on sociodemographic characteristics, medical data based on medical claims, and the results of national general health screening for the whole population of South Korea^37^. Sociodemographic characteristics include anonymized code for each individual, age, gender, socioeconomic variables, residential area, and household income level. Medical data in NHID include inpatient and outpatient usage, diagnostic codes by the International Classification of Diseases 10^th^ revision (ICD-10), treatment procedures, and prescription records. In addition, the national general health screening is provided free of charge for workplace subscribers and their dependents and all Koreans over the age of 40 at least biannually and the participation rate has been over 70% since 2011^37^. It includes information on health behaviors and general laboratory tests. Furthermore, the KNHIS has a registration program which offers additional coverage for cancer-related medical fees once diagnosis of malignancy is confirmed, resulting in a reliable method for identifying cancer patients using the NHID.

This study was approved by the Institutional Review Board (IRB) of the Yeouido St. Mary’s Hospital, The Catholic University of Korea, and was conducted according to the ethical principles outlined in the Declaration of Helsinki. Informed consent was not obtained, because anonymized and de-identified information was used for analyses.

### Study population and design

Subjects who were diagnosed with glaucoma between 2007 and 2016 who also received a national general health screening in the same year were assigned as the glaucoma group. Individuals under the age of 40 and those with less than 1 year follow up were excluded from the analysis. Glaucoma was defined based on ICD-10 code (H401). To enhance the validity of the diagnosis, those with at least 3 clinical records with glaucoma diagnosis during the index year were included in the study^38^.

Controls were 1:1 matched based on age and sex during the same period. We collected additional data on subjects’ household income (lowest quartile or the remaining quartiles), place of residence (urban or rural), smoking history (no, ex-smoker, or current smoker), comorbidities such as diabetes (E11-E14), hypertension (I10–13 and I15), and dyslipidemia (E78), using ICD-10 diagnostic codes, and the national general health screening results including body mass index, cholesterol level, systolic/diastolic blood pressure, and fasting glucose level. Presence of comorbidities was defined as diagnoses of the aforementioned codes within one year before the index date and prescription history of relevant medications.

To define individuals with various site-specific cancer, the ICD-10 diagnostic codes were used (Supplementary Table 1)^39,40^. To include only patients with newly-diagnosed cancer, we excluded those with prior diagnosis of cancer between 2002 and the index date. Each subject was followed up for development of cancer until 2017.

### Statistical analysis

To compare the population characteristics between cohorts, Student’s t-test and χ^2^ test were used to compare continuous and categorical variables, respectively. Cancer incidence rates were calculated per 1,000 individuals.

To investigate the association between glaucoma and incident cancer, we calculated the hazard ratios (HRs) and 95% confidence intervals (CIs) using the multivariate Cox regression analysis with multiple adjustments; the fully-adjusted model included matched variables (age and gender) as well as unmatched variables (diabetes, smoking history, and BMI). We used the SAS ver 9.4 (SAS Institute, Cary, NC, USA) with *P* values < 0.05 considered as statistically significant.

## Supplementary information

Supplementary Information.
