# Supplementary material for: Risk of cancer in patients with glaucoma: A nationwide population-based cohort study
Source: Sci Rep. 2020 May 18;10:8170. doi: 10.1038/s41598-020-65116-8 (PMC7235084; doi:10.1038/s41598-020-65116-8)

Risk of cancer in patients with glaucoma: A nationwide population-based cohort study

Younhea Jung, MD, PhD[a], Kyungdo Han, PhD[b], Kyung-sun Na, MD, PhD[a], Gee-hyun Kim, MD[a], Minji Ha, MD[a], Ji-Sun Paik, MD, PhD[a], Jung Il Moon, MD, PhD[a]

Supplementary Table 1. ICD-10 codes of specific cancers

Cancer	ICD-10 codes
Overall cancer	C
Oral	C00-C14
Esophagus	C15
Laryngeal	C32
Thyroid	C73
Stomach	C16
Colorectal	C18-21
Liver	C22
Pancreatic	C25
Biliary	C23, C24
Lung	C33, C34
Renal	C64
Bladder	C67
Cancer of central nervous system	C70-72
Leukemia	C91-95
Lymphoma	C81-86
Multiple myeloma	C90
Skin	C43
Prostate	C61
Testicular	C62
Breast	C50
Uterine cervical	C53
Uterine corpus	C54, C55
Ovarian	C56

ICD-10: International Classification of Diseases, Tenth Revision.

Supplementary Figure 1. Study population

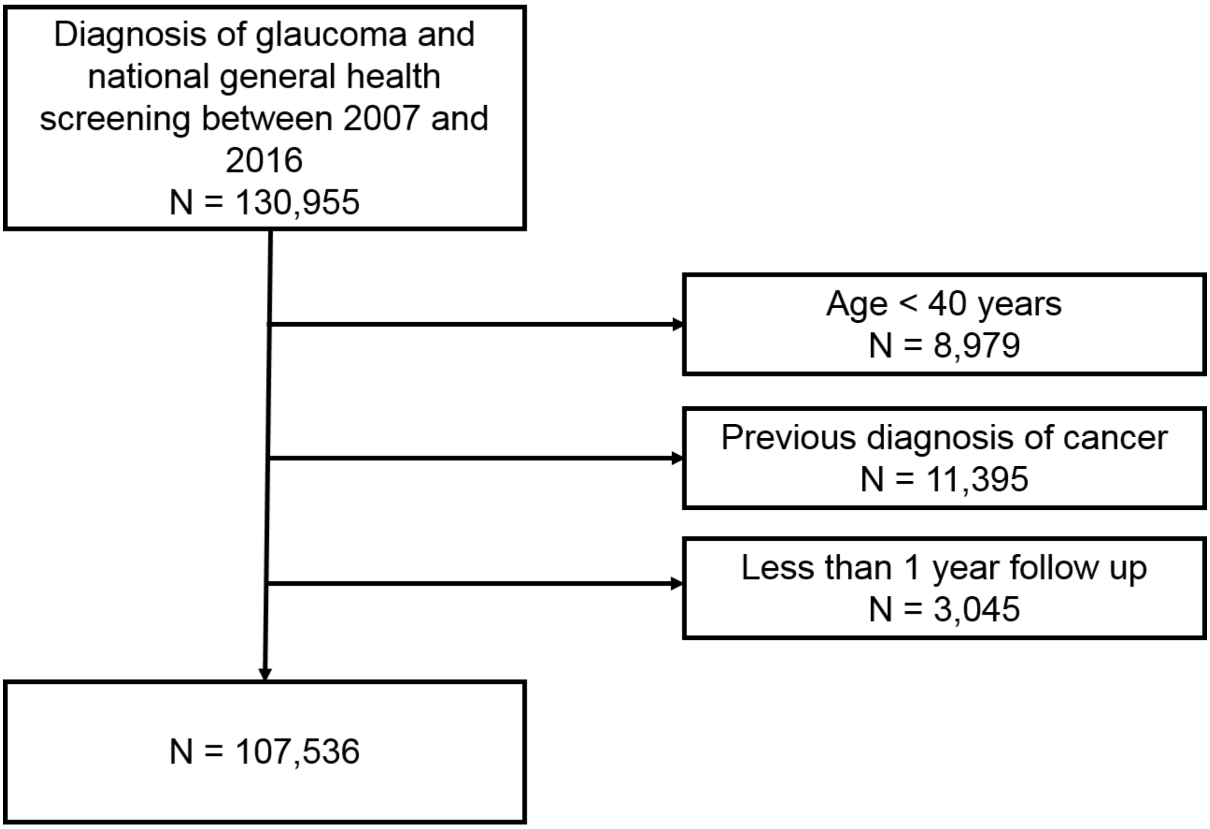

Supplement: Supplementary file 1 — Supplementary Information. [file 41598_2020_65116_MOESM1_ESM.pdf]
